# Smart Healthcare: The Role of Digital Health in Modern Medicine

**DOI:** 10.1002/hcs2.70019

**Published:** 2025-06-01

**Authors:** Nidha Shapoo, Naveed Shapoo, Abdul Rehman, Noella Boma

**Affiliations:** ^1^ Department of Medicine New York Medical College/Metropolitan Hospital New York USA; ^2^ Department of Anesthesia Sheikh Khalifa Medical City Ajman United Arab Emirates

**Keywords:** challenges, digital health, healthcare, implementation, innovations, solutions

## Abstract

Digital health is transforming healthcare by integrating advanced technologies to make healthcare more accessible, efficient, and personalized. From electronic health records, telemedicine, wearable devices, and artificial intelligence to the recent smart hospitals, digital health is improving patient care and outcomes while reducing healthcare costs. However, the integration of digital health faces several challenges, including data privacy, cybersecurity risks, and inequitable access to technology. This article provides an overview of the current state of digital health, key challenges in implementation, and potential solutions to maximize the benefits of digital health and ensure efficient, equitable, and patient‐centered healthcare in the future.

## Graphical abstract

Implementation of Digital Health. Innovations in Digital Health. Challenges in Digital Health.

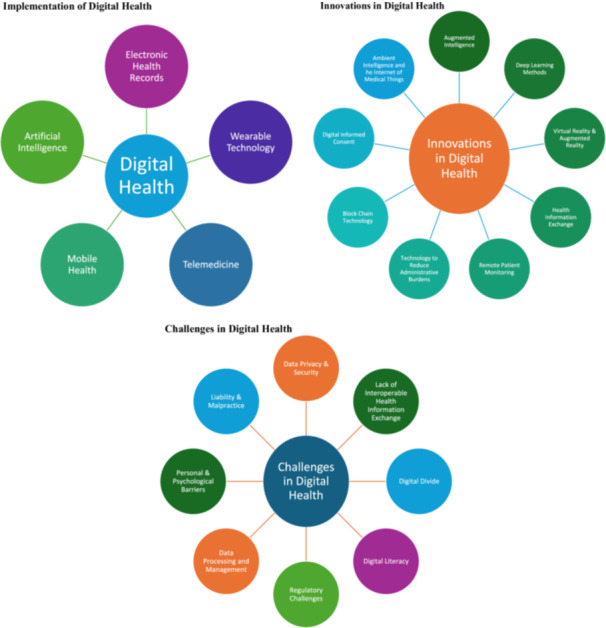

**Abbreviations**
AI: artificial intelligence
EHRs: electronic health records
IoMT: internet of medical things

## 1 Introduction

The swift progression of technology has revolutionized numerous sectors, and healthcare is no exception. Incorporating digital care into healthcare delivery has revolutionized disease management, especially in specific populations such as patients with cancer, patients with chronic illnesses, and older adults. Digital health, which encompasses many technologies designed to enhance health and healthcare delivery, has improved patient participation, augmented care coordination, and enabled remote monitoring [1].

The notion of digital health has progressed over several decades since the advent of electronic health records (EHRs) in the 1960s [2]. The advent of the internet in the late 20th century facilitated the development of advanced digital health solutions such as telehealth services and online patient portals. The surge of smartphones in the 21st century has significantly expedited the expansion of mobile health applications and allowed patients to conveniently oversee their health from their devices.

However, a core research problem persists: the integration and effectiveness of digital health technologies in real‐world clinical settings. Challenges include interoperability between electronic health systems, data privacy and security concerns, the digital divide that affects equitable access, and the need for evidence‐based validation of digital interventions. Addressing these issues is crucial for ensuring that digital health solutions are not only innovative but also practical, secure, and beneficial for patients and healthcare providers.

**Figure 1:**
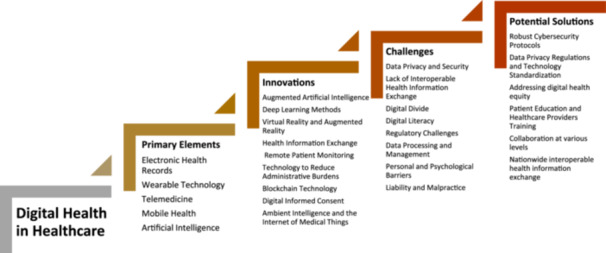
The primary elements, innovations, challenges, and potential solutions of digital health in healthcare.

This article examines the deployment of digital health solutions, emphasizes significant developments, and addresses the challenges in digital health by providing potential solutions for increasing the adoption of these technologies (Figure 1). Relevant stakeholders include healthcare professionals, medical researchers, hospital administrators and policymakers, technology developers in the pharmaceutical industry, insurance providers, and patients.

## 2 Implementation of Digital Health

Integrating technology into existing healthcare systems by implementing digital health solutions enhances patient care, streamlines operations, and improves health outcomes. The following are the primary elements of digital health implementation (Table 1).

**Table 1:** The primary elements of digital health in healthcare.

The primary elements of digital health in healthcare
1. Electronic health records
2. Wearable technology, e.g., fitness trackers, smartwatches, and insulin pumps
3. Telemedicine including teleoncology, teleradiology, telepathology, telepharmacology
4. Mobile health using mobile apps and sensors
5. Artificial intelligence

### 2.1 Electronic Health Records

EHRs facilitate digitizing patient records and have emerged as a standard instrument in healthcare environments. EHRs improve data management, enhance communication among healthcare providers, and increase patient safety by making information more accessible. EHRs enhance patient‐centered care by providing information including clinical and laboratory data regarding risk factors and allowing patients to monitor their health through digital applications, potentially with clinician supervision and support [3].

### 2.2 Wearable Technology

Wearable technology includes fitness trackers and smartwatches that monitor health metrics and deliver real‐time data to patients and healthcare providers. Wearable devices monitor patients' vital signs, sleep patterns, and physical activity and offer data that inform treatment decisions and support symptom management [4]. Specific wearable devices incorporate functionalities such as electrocardiogram monitoring and fall detection. This information can be communicated to healthcare providers for ongoing monitoring and prompt intervention. Researchers are investigating integrating augmented reality and artificial intelligence (AI) into wearable devices to enhance user experience and facilitate health prediction or personalized recommendations [5, 6]. Therapeutic devices, including insulin pumps, implantable cardiac pacemakers, and deep brain stimulation devices, are utilized for continuous monitoring and treatment [7].

### 2.3 Telemedicine

The coronavirus disease 2019 pandemic expedited the implementation of telemedicine, which allows healthcare providers to conduct virtual consultations. This method enhances healthcare accessibility, particularly for individuals in isolated regions or those experiencing mobility challenges. Telehealth services enable patients with chronic conditions to conduct regular check‐ups remotely by enhancing access to care and facilitating continuous management of their health issues. Teleoncology has emerged as a critical instrument in the field of oncology. Virtual consultations allow oncologists to assess patients, consider treatment alternatives, and oversee side effects from a distance. Hospital‐based telemedicine is rapidly expanding in two domains: stroke management and intensive care unit services. Research indicates that practical imaging enables the execution of remote high‐quality stroke examinations. Ancillary telemedicine services, including teleradiology, telepathology, and telepharmacology, are experiencing significant growth [8].

### 2.4 Mobile Health

Mobile health applications allow patients to oversee their health using mobile apps and sensors independently or in conjunction with clinical team members and support from friends and family. Mobile health encompasses the use of devices that measure novel biomarkers and consumer versions of traditional clinical equipment, such as blood pressure cuffs and spirometers, allowing individuals to collect measurements at their convenience, independent of clinical indication. These applications enhance patient engagement by providing medication reminders, monitoring treatment schedules, and delivering educational resources specific to the disease and treatment options [9].

### 2.5 Artificial Intelligence

AI has emerged as a transformative force in healthcare that provides innovative solutions for enhancing the accuracy of disease diagnosis, improving treatment strategies, and predicting patient prognosis [10, 11]. AI tools have enhanced workflow efficiency, reduced patient turnaround time, and increased the accuracy and reliability of patient data. AI has demonstrated efficacy in interpreting diverse medical images, including pathology slides, various radiographs, retinal scans, and images of skin lesions, for disease diagnosis. Numerous studies indicate that AI can analyze these images with accuracy that is comparable with or surpasses that of experienced clinicians [12]. AI exhibits significant sensitivity and specificity in detecting early‐stage cancers and frequently surpasses the performance of human radiologists [13]. AI has shown potential in diagnosing conditions that include prostate cancer (notably Gleason scoring) and lung, colon, breast, and skin cancers [14, 15, 16]. AI holds significant potential for analyzing extensive patient data in cancer genomics, potentially yielding diagnostic, prognostic, and therapeutic insights [17]. AI can support treatment planning and forecast treatment failure in radiation therapy [18]. Furthermore, AI can assist in end‐of‐life decisions, including resuscitation and the initiation of mechanical ventilation [19]. AI technologies are incorporated into wearable devices and mobile health applications to facilitate the continuous monitoring of patients with chronic conditions. These tools facilitate the timely adjustment to treatment plans by analyzing real‐time data and alerting healthcare providers when intervention is necessary [20, 21].

## 3 Innovations in Digital Health

Digital health has experienced numerous innovations that enhance patient care and improve healthcare delivery (Table 2). The following are noteworthy examples of digital health:

**Table 2:** Innovations in digital health.

Innovations in digital health
1. Augmented artificial intelligence
2. Deep learning methods
3. Virtual reality and augmented reality
4. Health information exchange
5. Remote patient monitoring
6. Technology to reduce administrative Burdens like advanced EHRs and speech recognition
7. Blockchain technology
8. Digital informed consent
9. Ambient intelligence and the Internet of Medical Things.

### 3.1 Augmented Artificial Intelligence

Augmented intelligence emphasizes the assistive role of AI by highlighting that AI design enhances rather than replaces human intelligence. Augmented intelligence can assist in various domains in the future: supporting patient triage according to symptom severity, utilizing electrocardiograms to assess valvular disease progression, automating real‐time predictions of optimal therapy for individual patients, analyzing multi‐modal wearable data to predict, detect, and classify epileptic seizures, automatically identifying the risk of postpartum hemorrhage hours in advance for all patients, conducting noninvasive genomic analysis of cancer using imaging phenotypes, and predicting the risk of acute illnesses (e.g., pneumonia) earlier than with the current practice of X‐ray analysis. Augmented intelligence can facilitate staff management, education, and research and ensure billing accuracy and quality enhancement [22, 23].

### 3.2 Deep Learning Methods

Deep learning methods are representation learning algorithms that utilize multiple representation levels. These levels are achieved by composing simple, nonlinear modules and transforming the representation from one level to a higher, more abstract level [24]. Deep learning can enhance healthcare with its superior performance, end‐to‐end learning framework that incorporates feature learning, and ability to manage complex and multi‐modal data. Deep learning has been utilized to analyze aggregated EHRs that encompass both structured data (e.g., diagnoses, medications, laboratory tests) and unstructured data (e.g., free‐text clinical notes) [25]. Deep learning can predict diseases based on patient clinical status [26] and is important in the early detection of cancer. Uhm et al. employed deep learning models to distinguish between the histological subtypes of renal tumors by categorizing tumors as malignant or benign [27]. Deep learning algorithms have demonstrated significant accuracy in early lung cancer diagnosis [28].

### 3.3 Virtual Reality and Augmented Reality

Virtual and augmented reality are innovative healthcare technologies that are utilized for medical training, patient education, therapeutic interventions, and surgical planning [29]. Virtual reality can reduce stress, pain, and anxiety in critical care settings while facilitating coordination, mobilization, and physical and mental rehabilitation [30, 31]. Virtual and augmented reality can assist in the planning and execution of intricate cardiovascular procedures [32].

### 3.4 Health Information Exchange

Health information exchange platforms enable the secure transfer of health and administrative data between diverse healthcare providers. This interoperability improves care coordination, minimizes test duplication, and supports providing high‐quality care [33]. Health information exchange platforms provide clinicians with a complete picture of patient health status at the point of care to ensure quality and patient safety. Health information exchange can also reduce medical costs, duplicative utilization, and the administrative burden on patients and clinicians by ensuring seamless data flow across the continuum of care [34].

### 3.5 Remote Patient Monitoring

Remote patient monitoring technologies enable healthcare providers to track patients' health metrics beyond the clinical environment. Continuous monitoring decreases hospital readmissions and enhances disease management by facilitating early intervention. Remote patient monitoring is primarily beneficial for patients with chronic illnesses and those who require complex care, such as individuals with cancer [35].

### 3.6 Technology to Reduce Administrative Burden

By alleviating the administrative burden by streamlining documentation, coding, and prior authorizations, advanced EHRs enhance professional satisfaction and mitigate physician burnout by allowing physicians to concentrate on addressing patients' medical needs instead of managing excessive paperwork [36]. Speech recognition software facilitates the conversion of voice commands into text, allowing clinicians to reduce time spent on documentation and increase patient interaction [37].

### 3.7 Blockchain Technology

Blockchain provides a secure and decentralized framework for managing health information and improving data integrity and security. This innovation enhances patient privacy, health data analytics, biomedical research, and electronic medical records. Blockchain can safeguard healthcare data against risks of data loss, corruption, and security breaches, including ransomware attacks [38].

### 3.8 Digital Informed Consent

Informed consent guarantees that research participants are adequately informed about the specifics of a study and voluntarily participate. The objective of informed consent is to provide prospective participants with adequate information that is presented clearly and comprehensibly, enabling them to make an informed decision regarding study participation. Although informed consent is vital to patient engagement, formalizing the process has introduced particular challenges. Innovative methods, including video consent, smartphone consent, and digital informed consent, are being employed to address these challenges [39].

### 3.9 Ambient Intelligence and the Internet of Medical Things (IoMT)

Ambient intelligence and the IoMT represent a paradigm shift toward intelligent healthcare and smart hospitals. Integrating advanced sensors, actuators, and AI with ambient intelligence technology facilitates remote patient monitoring, telemedicine, virtual consultations, clinical decision‐making, assisted living for older adults, and many more opportunities. The IoMT has led to the generation of EHRs, allowing the seamless sharing of patient data among healthcare providers and enabling collaborative care and proactive patient monitoring [40, 41, 42]. Smart hospitals use the IoMT to track a patient's condition, improve patient outcomes, manage hospital assets, and optimize resource allocation.

## 4 Challenges in Digital Health

Although digital health offers numerous advantages, various challenges impede its broad adoption and effective implementation (Table 3).

**Table 3:** Challenges in digital health.

Challenges in digital health
1. Data privacy and security
2. Lack of interoperable health information exchange
3. Digital divide
4. Digital literacy
5. Regulatory challenges
6. Data processing and management
7. Personal and psychological barriers
8. Liability and malpractice

### 4.1 Data Privacy and Security

Data privacy and security are one of the most prominent digital health challenges. In 2021, over 22.6 million patients were affected by healthcare‐related data breaches. Collecting and managing sensitive health data is associated with considerable privacy and security concerns [43]. Compliance with regulations such as HIPAA is essential for the protection of sensitive information. Digital health technologies can influence patient autonomy, particularly regarding data sharing and consent [44]. According to the HIPAA Journal, the Office for Civil Rights has reported an upward trend in data breaches over the past 14 years. In 2023, the Office for Civil Rights reported a 239% increase in hacking‐related data breaches between 1 January 2018 and 30 September 30 2023 and a 278% increase in ransomware attacks over the same period. While hacking accounted for 49% of all reported breaches in 2019, 80% of data breaches were attributed to hacking incidents in 2023. Implementing effective cybersecurity protocols may be challenging for healthcare facilities with limited resources, whose systems remain exposed to cyberattacks and breaches. For example, India's healthcare system faced 1.9 million cyberattacks in 2022 [45]. In 2023, the average cost of a healthcare data breach was the highest across industries at $10.93 million per incident, according to the IBM Cost of a Data Breach Report 2023.

### 4.2 Lack of Interoperable Health Information Exchange

The lack of interoperability among healthcare facilities and systems can lead to insecure data transfers and increase errors and risks [46]. The Office of the National Coordinator for Health tracks hospitals' overall engagement in four domains of interoperable exchange (send, receive, find, and integrate) to measure the effects of federal policy and industry efforts on interoperability. In 2023, 70% of nonfederal acute care hospitals engaged in all of the domains of interoperable exchange; among these hospitals, 43% routinely engaged in interoperable exchange, whereas 27% sometimes engaged in interoperable exchange. Less‐resourced hospitals (i.e., small, rural, critical access, or independent hospitals) engaged less frequently in interoperable exchange compared with their more‐resourced counterparts [47].

### 4.3 Digital Divide

Disparities in access to technology have resulted in inequalities within healthcare systems that significantly affect access to care and patient outcomes. Disparities can be observed in socioeconomic status, racial and ethnic inequities, geographic location, age, and education [48]. Digital health technology interventions are more effective for individuals who are already in advantageous positions. This phenomenon is recognized within public health as intervention‐generated inequalities [49]. According to the United Nations Educational, Scientific and Cultural Organization, approximately 45.2% of households worldwide lack internet access. Individuals who earn below the $30,000 income threshold are more likely to use phones rather than tablets or laptops for internet access because of limited broadband availability [50]. Rural adults reported lower access to telehealth services than their non‐rural counterparts, and 75% expressed a willingness to use telehealth services—similar to non‐rural adults [51]. After adjusting for median household income, broadband access in predominantly Black and Hispanic neighborhoods was 10%–15% lower than in predominantly White or Asian neighborhoods [52]. Despite a recent increase in the adoption rate of digital technology among older adults, the digital divide continues to present a significant global challenge for older adults, especially those with low socioeconomic status and from racial and ethnic minority groups [53].

### 4.4 Digital Literacy

Educational disparities lead to differential capabilities among children and adults in utilizing digital technologies and comprehending associated risks. Digital literacy refers to the technical skills required to utilize and access the internet and the ability to critically and confidently interact with the online environment. Seventy‐eight percent of individuals with college degrees use digital health tools compared with only 39% with a high school diploma or less. Digital health literacy has been recognized as a significant factor among the primary social determinants of health given its impact on the broader social determinants of health [54, 55].

### 4.5 Regulatory Challenges

The dynamic characteristics of digital health technologies may complicate the regulatory approval process from both product (adaptability, variability, variety, novelty, and accessibility) and industry‐structural (new entrants, changing roles of actors, and new delivery models) perspectives. Regulatory and reimbursement policy uncertainties regarding digital health services can impede their adoption. The lack of comprehensive policy frameworks and regulatory mechanisms, outdated guidelines, and legal ambiguities has created gaps [56]. Weak or absent regulations regarding insurance coverage for telemedicine and digital health services further limit financial accessibility. Clear and consistent guidelines are essential for ensuring the safety and efficacy of digital health solutions [57].

### 4.6 Data Processing and Management

The substantial volume of IoMT data demands effective pattern analysis to ensure informed decision‐making. Whereas traditional methods rely on manual observation using self‐reporting tools such as questionnaires and interviews, big data platforms provide automated frameworks such as MapReduce and Hadoop for parallel and distributed analysis. Tools such as Cascading, Pig, and Hive facilitate handling interrelated data groups. However, selecting the most suitable framework poses a challenge [42].

### 4.7 Personal and Psychological Barriers

These barriers include healthcare professionals' resistance to change; challenges in comprehending technology; perceptions of diminished human interaction; technophobia; variations in age, education level, and professional experience; low literacy; inadequate writing skills; linguistic characteristics; adherence behavior; and apprehension regarding the use of health technology. The apprehension of increased workload and modified workflow is an additional barrier that affects the quality of care [58].

### 4.8 Liability and Malpractice

Healthcare providers' increasing reliance on digital health tools to support diagnosis and treatment decisions raises pertinent questions regarding liability. Establishing accountability for misdiagnosis or treatment failure associated with digital health technologies can complicate malpractice claims [59].

## 5 Potential Solutions to Improve the Effectiveness of Digital Health

Addressing challenges is essential for maximizing the potential of digital health technologies and enhancing healthcare accessibility to improve patient outcomes. The following are potential solutions to address these challenges (Table 4).

**Table 4:** Potential solutions for improving the effectiveness of digital health.

Potential solutions to improve the effectiveness of digital health
1. Robust cybersecurity protocols
2. Data privacy regulations and technology standardization
3. Addressing digital health equity by improving technology access, training programs, and equal health services reimbursement
4. Patient education and healthcare providers training
5. Collaboration at various levels, e.g., regulatory bodies, policymakers, technology developers, healthcare providers, and patients
6. Nationwide interoperable health information exchange

### 5.1 Robust Cybersecurity Protocols

Enhancing cybersecurity protocols, such as ensuring end‐to‐end encryption, access controls, biometric authentication, and regular security audits, is critical for protecting sensitive health information and fostering patient trust. Hospitals should adopt zero‐trust security models to prevent unauthorized data access. To ensure trust and security in digital health, more potent encryption methods, stricter compliance with global data protection laws, improved interoperability frameworks, and increased transparency in data usage are required to address these challenges [44, 56].

### 5.2 Data Privacy Regulations and Technology Standardization

Implementing regulations to ensure stricter compliance with HIPAA and other healthcare data protection laws is crucial for preventing breaches. Standardized data exchange protocols, including HL7 and FHIR, enhance communication among digital health systems and improve care coordination. Establishing rigorous standards for developing and validating digital health solutions enhances quality. Promoting clinical trials and studies can yield evidence of efficacy and enhance credibility [46, 56].

### 5.3 Addressing Digital Health Equity

(1) Technology access and training programs: Initiatives that enhance technology access in rural and underserved communities, including initiatives that provide subsidized devices or internet connectivity, can effectively bridge the digital divide and promote equitable access to digital health solutions. Community‐based digital health training can be provided for low‐income and minority groups through workshops on telemedicine and patient portals [49]. (2) Equal health services reimbursement: Insurance providers such as Medicare, Medicaid, and private insurers can provide equal reimbursement for in‐person and telehealth visits [56].

### 5.4 Patient Education and Healthcare Provider Training

Providing education and training for patients and healthcare providers can enhance digital literacy and improve user engagement with digital health tools. User‐friendly interfaces can facilitate adoption by ensuring that digital health platforms support multiple languages and voice navigation. Regularly offering incentives to patients who use wearable devices can enhance the adoption of digital health. Involving healthcare providers in designing and implementing digital health solutions ensures alignment with existing workflows. Continuous training and support enhance integration processes. Early education about telehealth services can be provided by integrating digital health training into medical school curricula [54, 56].

### 5.5 Collaboration at Various Levels

Collaborating with regulatory bodies to establish clear guidelines for digital health solutions facilitates the trouble‐free navigation of compliance requirements by developers. Efficient approval processes can foster innovation. Encouraging collaboration among healthcare providers, technology developers, policymakers, and patients increases the development of more effective and user‐centered digital health solutions. Multi‐stakeholder partnerships facilitate innovation and allow the tackling of common challenges [56, 58].

### 5.6 Nationwide Interoperable Health Information Exchange

Although the number of hospitals that routinely engage in interoperable exchange has increased by 54% since 2018, an ongoing need remains for comprehensive engagement in interoperability across the healthcare continuum to improve nationwide interoperability. To address these gaps, initiatives such as the Trusted Exchange Framework and Common Agreement that are designed to improve connectivity and minimize barriers to data exchange are steps forward in facilitating equitable access to and use of patient health information [60].

## 6 Conclusion

By offering innovative solutions to improve patient care and healthcare delivery, digital health has become a transformative force in the healthcare sector. As the field evolves, addressing challenges such as data privacy, the digital divide and literacy, and the interoperability of healthcare systems is essential for maximizing the potential of digital health technologies. Implementing equitable policies in digital health, data privacy regulations, and standardized protocols and enhancing patient and healthcare education can make digital health more accessible, secure, and effective for diverse populations. Overall, digital health is expected to empower patients and enhance healthcare accessibility worldwide by continuing to bridge the gaps in healthcare delivery. A balanced approach that includes collaboration with various stakeholders, including policymakers, healthcare providers, and technology companies, is the key to success in driving meaningful change in digital healthcare adoption.

## Author Contributions

**Nidha Shapoo:** conceptualization (equal), data curation (equal), formal analysis (equal), methodology (equal), writing – original draft (equal), writing – review and editing (equal). **Naveed Shapoo:** data curation (equal), resources (equal), writing – original draft (equal), writing – review and editing (equal). **Abdul Rehman:** resources (equal), writing – original draft (equal). **Noella Boma:** conceptualization (equal), supervision (equal), writing – review and editing (equal).

## Ethics Statement

The authors have nothing to report.

## Consent

The authors have nothing to report.

## Conflicts of Interest

The authors declare no conflicts of interest.

## Data Availability

Data openly available in a public repository that issues datasets with DOIs.
